# Lack of unique neuropathology in amyotrophic lateral sclerosis associated with p.K54E angiogenin (*ANG*) mutation

**DOI:** 10.1111/nan.12007

**Published:** 2012-12-10

**Authors:** J Kirby, J R Highley, L Cox, E F Goodall, C Hewitt, J A Hartley, H C Hollinger, M Fox, P G Ince, C J McDermott, P J Shaw

**Affiliations:** * Academic Unit of Neurology, Department of Neuroscience, Sheffield Institute for Translational Neuroscience (SITraN), University of SheffieldSheffield, UK; † Academic Unit of Pathology, Department of Neuroscience, Sheffield Institute for Translational Neuroscience (SITraN), University of SheffieldSheffield, UK

**Keywords:** amyotrophic lateral sclerosis, angiogenin, glial inclusions, intranuclear inclusions, neuronal inclusions, neuropathology

## Abstract

**Aims:**

Five to 10% of cases of amyotrophic lateral sclerosis are familial, with the most common genetic causes being mutations in the *C9ORF72*, *SOD1*, *TARDBP* and *FUS* genes. Mutations in the angiogenin gene, *ANG*, have been identified in both familial and sporadic patients in several populations within Europe and North America. The aim of this study was to establish the incidence of *ANG* mutations in a large cohort of 517 patients from Northern England and establish the neuropathology associated with these cases.

**Methods:**

The single exon *ANG* gene was amplified, sequenced and analysed for mutations. Pathological examination of brain, spinal cord and skeletal muscle included conventional histology and immunohistochemistry.

**Results:**

Mutation screening identified a single sporadic amyotrophic lateral sclerosis case with a p.K54E mutation, which is absent from 278 neurologically normal control samples. The clinical presentation was of limb onset amyotrophic lateral sclerosis, with rapid disease progression and no evidence of cognitive impairment. Neuropathological examination established the presence of characteristic ubiquitinated and TDP-43-positive neuronal and glial inclusions, but no abnormality in the distribution of angiogenin protein.

**Discussion:**

There is only one previous report describing the neuropathology in a single case with a p.K17I *ANG* mutation which highlighted the presence of eosinophilic neuronal intranuclear inclusions in the hippocampus. The absence of this feature in the present case indicates that patients with *ANG* mutations do not always have pathological changes distinguishable from those of sporadic amyotrophic lateral sclerosis.

## Introduction

Amyotrophic lateral sclerosis (ALS) is an adult onset neurodegenerative disease characterized by degeneration of motor neurones in the cerebral cortex, brainstem and spinal cord. It results in progressive muscular weakness, wasting and paralysis that typically causes death within 3–5 years of onset 1. Five to 10% of ALS cases are familial and causative mutations have been identified in several genes, the most common being *C9ORF72*, *SOD1*, *TARDBP* and *FUS* 2–7. An association between the angiogenin gene (*ANG*), located on chromosome 14q11.2, and ALS was originally demonstrated by Greenway and colleagues. They identified that the G allele of the synonymous rs11701 single nucleotide polymorphism (SNP) was overrepresented in Scottish and Irish ALS cases compared with controls 8. Subsequent screening of the coding region of the *ANG* gene in 1629 ALS cases identified seven missense mutations in 15 ALS patients, including four familial and 11 sporadic cases 9. Mutations in *ANG* have subsequently been associated with ALS in nine different populations 10–18. To date 20 different mutations have been identified in ALS patients (Table 1), with *ANG* mutations accounting for 1.2–2.6% of familial ALS (FALS) cases and 0.35–0.8% of sporadic ALS (SALS) cases.

**Table 1:** *ANG* mutations reported in ALS cases to date

Population	Number of ALS patients (SALS/FALS)	Amino acid substitutions	Reference/comments
Scottish	398 (364/34)	p.Q12L (×2)	[9] *p.K17I also identified in one control
Irish	293 (262/31)	*p.K17I (×2)	
Swedish	434 (334/100)	p.K17E (×2)	
USA (Boston)	360 (277/83)	p.R31K	
England	144 (133/11)	p.C39W (×2)	
		p.K40I (×3)	
		p.I46V (×3)	
North American	298 (not given)	p.P(-4)S	[10]
		p.K17I	
		p.S28N	
		p.P112L	
French	855 SALS	p.I46V (×2)	[17]
		p.R121H	
German	581 SALS	p.F(-13)L	[16] *p.K17I also found in two controls
		*p.K17I	
		p.I46V	
		p.K54E	
Dutch	39 FALS index cases	*p.K17I	[13] *p.K17I in six affected and one unaffected family member
Italian	737 (605/132)	p.M(-24)I	[11] *p.I46V also found in four controls
		p.F(-13)S	
		p.P(-4)S	
		*p.I46V (×6)	
		p.V113I (×2)	
		p.H114R	
Italian	262 (215/12; 35 unknown)	*p.I46V (×1)	[12] *p.I46V also found in two controls
South Italian	163 (155/8)	p.M(-24)I	[15] *p.I46V also found in five controls
Italian	210 SALS	None	[14]
French	162 FALS index cases	p.K17I (×2)	[40] p.K17I also carried FUS p.R521C, p.K54E also carried FUS p.R521S
		p.K54E	
		p.R121H	
Italian	1	p.R145C	[41] Also carried SOD1 p.G93D
Belgian	310 SALS	p.M(-24)I (×2)	[18] *p.K17I and p.I46V were observed in all populations in ALS and
Dutch	980 (941/39)	p.F(-13)L	controls at comparable frequencies
Swedish	277 SALS	p.F(-13)S	
		p.G(-10)D	
		p.P(-4)Q	
		p.P(-4)S (×2)	
		p.Q12L (×2)	
		p.K17E (×2)	
		p.S28N	
		p.R31K	
		p.C39W (×2)	
		p.K40I (×6)	
		p.K54E	
		p.T80S	
		p.F100I	
		p.P112L	
		p.V113I (×3)	
		p.H114R	
		p.R121H	

* Amino acid changes also found in controls.

ALS, amyotrophic lateral sclerosis; SALS, sporadic ALS; FALS, familial ALS.

Mature ANG is a 14 kDa, 123 amino acid, secreted protein predominantly produced by the liver. It is a member of the pancreatic ribonuclease superfamily and was originally identified as a potent factor stimulating new blood vessel growth secreted by tumour cells 19. Exogenous ANG binds to a 170 kDa receptor on the surface of endothelial cells and is transported into the cell by receptor-mediated endocytosis. Once inside the cell ANG is rapidly translocated to the nucleus where it accumulates inside the nucleolus. There it binds to ribosomal DNA and stimulates ribosomal RNA synthesis, a rate-limiting step in ribosome biogenesis which is required for active cell proliferation 20. Both the ribonuclease activity of ANG and its translocation to the nucleus are essential for its angiogenic and cell proliferative activity. ANG also has a role in the cytoplasm where it has been shown to cleave transfer RNAs in response to cellular oxidative stress resulting in an important alternative, eIF2α-independent pathway of translational inhibition 21. ANG is widely expressed throughout the adult human nervous system, is present inside motor neurones and has been shown to be directly neuroprotective 10,22.

The aim of this investigation was to screen the coding region of *ANG* for mutations in a large cohort of patients from Northern England. We have identified a single SALS case with a p.K54E mutation; both the clinical and neuropathological findings of the individual are presented.

## Materials & methods

### Genetic screening

DNA samples were obtained from blood taken from patients attending the Motor Neurone Disorders Clinic at the Royal Hallamshire Hospital in Sheffield, England, and from the Sheffield Brain Tissue Bank, which contains *post mortem* tissue donated by ALS patients between 1989 and 2012. In total, 517 cases were screened, and these comprised the following groups of patients: 433 ALS (30 FALS), 36 progressive muscular atrophy, 11 progressive bulbar palsy, nine primary lateral sclerosis, 10 ALS plus dementia and 18 atypical ALS. None of these cases were positive for *SOD1*, *TARDBP* or *FUS* mutations; however, subsequent screening of this cohort identified 13 FALS and 16 SALS with expansions in the *C9ORF72* gene. Control DNA samples from 278 healthy age- and sex-matched individuals were obtained from blood samples donated by unrelated partners and carers of ALS patients, and from volunteers donating blood for transfusion at National Blood Service sessions in Sheffield. All cases and controls were of White Caucasian ethnicity. Approval for the use of DNA samples was obtained from the local research ethics committee and donor consent was obtained.

Genomic DNA was extracted from whole blood and fresh frozen central nervous system tissue using the Nucleon BACC Genomic Extraction Kit and Soft Tissue DNA Extraction Kit, respectively (Tepnel, UK). The *ANG* gene was amplified from genomic DNA by PCR using standard methods and published primer sequences 9. PCR products were sequenced using BigDye Terminator v3.1 (Life Technologies, Paisley, UK) according to manufacturer's instructions. Sequencing products were read on a 3730 DNA Analyser (Life Technologies) and chromatographs were aligned to the available reference sequence (NM_001145). Residues were numbered in accordance with the nomenclature recommended by the Human Genome Variation Society (http://www.hgvs.org). Controls were screened for the c.232A>G substitution by performing restriction digests of the *ANG* PCR products with *Taq*^α^I using standard methods. Mutant sequences yield two products at 287 bp and 263 bp, whereas the wild-type sequence remains uncut (550 bp).

### Pathology

An autopsy was performed with the consent of the next of kin. Representative blocks of brain, spinal cord and skeletal muscle were taken and fixed in formalin. They were then embedded in paraffin wax, sectioned and stained for haematoxylin and eosin and luxol fast blue. Immunohistochemistry (IHC) for FUS, TDP-43, p62, CD68, ANG, α-actinin-2 and α-smooth muscle actin was performed on 5 μm sections (Table 2). This study used the same antibody for ANG (MANG-1) as was employed in the existing neuropathologically characterized case report 23.

**Table 2:** Antibodies used in immunohistochemical studies

Antibody	Isotype	Dilution	Antigen retrieval	Incubation	Source
TDP-43	Polyclonal rabbit	1:200	Microwave 10 min trisodium citrate buffer pH 6.5	60 min at room temperature	Proteintech (Manchester, UK)
FUS/TLS	Polyclonal rabbit	1:100	Microwave for 20 min 1 mM EDTA pH 8.0	48 h at 4°C	Novus (Cambridge, UK)
p62 (3/P62 Lck ligand)	Polyclonal mouse	1:200	Microwave 10 min trisodium citrate buffer pH 6.5	60 min at room temperature	BD Transduction Laboratories (Oxford, UK)
Angiogenin (MANG-1)	Mouse monoclonal	1:750	Microwave 10 min trisodium citrate buffer pH 6.5	30 min at 37°C	AbD Serotec (Kidlington, UK)
α-smooth muscle actin (1A4)	Mouse monoclonal	1:75	Dako ENVISION™ FLEX antigen retrieval solution, low pH, 20 min at 97°C	60 min at room temperature	Dako (Ely, UK)
α-actinin-2	Polyclonal rabbit	1:50	Pressure cooker in EDTA pH 8	30 min at 37°C	Lifespan Biosciences (Stoke by Clare, UK)
CD68 (PGM1)	Mouse monoclonal	1:50	Trypsin bath at 37°C 10 min, pH 7.8	30 min at room temperature	Dako (Ely, UK)

## Results

### Genetic screening

**Figure 1:**
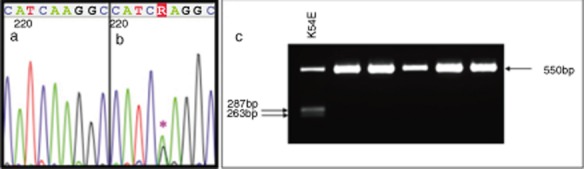
The p.K54E mutation identified in *ANG*. The non-synonymous A>G nucleotide substitution at position c.232 gives rise to the amino acid substitution, p.K54E. Chromatograms are shown for wild-type sequence (**a**), and the heterozygous c.232A>G case (**b**). Screening for this change in control samples was conducted by digestion of the *ANG* PCR product with *Taq*^α^I. Presence of the G allele introduces a *Taq*^α^I digest site, resulting in the production of 287 bp and 263 bp fragments from the 550 bp PCR product (**c**).

**Figure 2:**
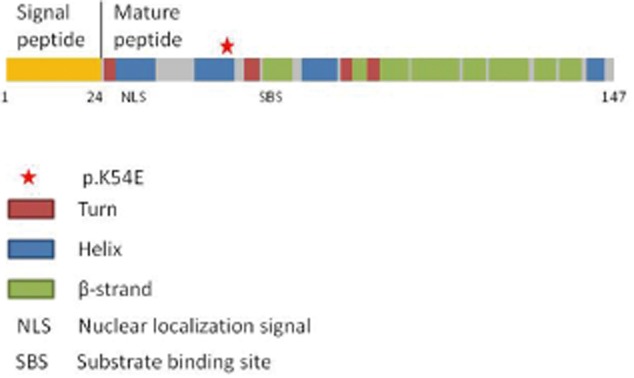
Schematic diagram of the ANG protein and location of the p.K54E mutation. The protein domains and secondary structure are derived from information provided on the ANG protein (P03950) found on the UniProt database.

Screening of the entire *ANG* coding region in the 516 cases identified a single heterozygous c.232A>G change in one patient with SALS (Figure 1). This change results in the substitution of lysine for glutamate at residue 54, p.K54E. This substitution was not detected in 278 neurologically normal controls, screened using the *Taq*^α^I digest (Figure 1). *In silico* analysis using SIFT 24 predicted that this mutation adversely affects the protein function, while I-Mutant 2.0 (http://folding.uib.es/i-mutant/i-mutant2.0.html) predicted decreased stability of the encoded protein. The amino acid change is located within the second helix of the mature ANG protein (Figure 2). This patient was negative for *SOD1*, *TARDBP* and *FUS* mutations, as well as negative for the *C9ORF72* expansion.

### Clinical findings

The individual in whom the p.K54E mutation was identified was a Caucasian male who presented at the age of 49 years with a 2-month history of lower limb weakness. He had initially noticed his right leg giving way and unsteadiness of gait. This had progressed over a few weeks to include right-sided foot drop. He had no significant prior medical history and no family history of neurological disease.

Initial neurological examination revealed no abnormalities in the cranial nerve territory. Widespread fasciculations were evident in the muscles of all four limbs. In the upper limbs tone and power were normal bilaterally and the reflexes were pathologically brisk. In the lower limbs, muscle tone was normal. Wasting of the right quadriceps muscle was observed and power was reduced bilaterally in hip flexion and ankle dorsiflexion. Knee jerks were brisk, ankle jerks were depressed and the plantar responses were flexor. No abnormalities of sensation were detected.

Routine laboratory investigations revealed only a slightly elevated creatine kinase level of 557 IU/l (reference range 25–190 IU/l). Cerebrospinal fluid (CSF) protein and cytology were normal. Magnetic resonance imaging (MRI) of the brain and spine was normal apart from multiple wedge fractures of thoracic and lumbar vertebrae without cord compression. Dual energy X-ray absorbance (DXA) scanning showed only vertebral osteopenia. A computerized tomography (CT) body scan performed did not identify any malignancy but did identify extensive bilateral pulmonary emboli for which the patient was prescribed warfarin. Respiratory function testing was normal at presentation. Neurophysiological examination revealed normal motor and sensory nerve conduction. Sensory nerve action potentials (SNAP) from both sural nerves were reduced. Motor studies in the tibial and peroneal nerves demonstrated reduced compound muscle action potentials (CMAP). Electromyography (EMG) in multiple upper and lower limb muscles revealed neurogenic changes compatible with anterior horn cell dysfunction. EMG of the tongue and paraspinal muscles was normal. A sural nerve biopsy was normal.

The patient's symptoms progressed rapidly resulting in severe bilateral lower limb weakness and followed by progressive weakness in the upper limbs. Repeat neurophysiology 6 weeks after the initial studies showed worsening motor axonal loss in all four limbs. He subsequently presented acutely to hospital with an episode of acute respiratory muscle weakness and died 6 days later from respiratory failure, 5 months after symptom onset. At this terminal stage of the disease there was no evidence of bulbar involvement and no cognitive impairment. The final clinical diagnosis was limb onset ALS, classified as probable ALS by the El-Escorial criteria.

### Pathology findings

**Figure 3:**
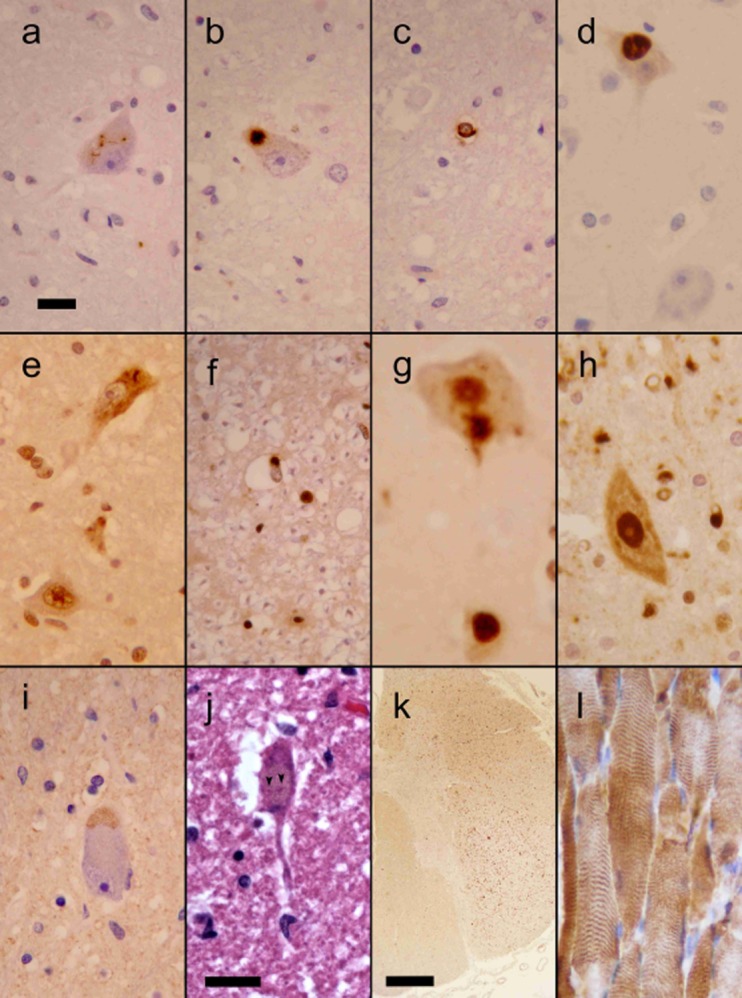
Immunohistochemistry of sporadic amyotrophic lateral sclerosis (SALS) case with p.K54E *ANG* mutation. Images show p62 (**a**–**d**), TDP-43 (**e**–**g**), FUS (**h**), angiogenin (**i**), H&E (**j**), CD68 (**k**) and α2 actinin (**l**) showing: neuronal (**a**, **b**, **d**, **e** and **g**) and glial (**c** and **f**) cytoplasmic inclusions in the spinal cord; neuronal cytoplasmic inclusions in the motor cortex (**d** and **g**); normal, predominantly nuclear labelling of FUS in the spinal cord (**h**), granular cytoplasmic staining in a motor neurone with the appearance of lipofuscin (**i**); Bunina bodies (arrowheads; **j**); a microglial reaction that is most marked in the lateral descending tract of the spinal cord and least marked in the dorsal columns (**k**); normal labelling of Z-disc in skeletal muscle by α2 actinin (**l**). Scale: **a**–**i** and **l**, bar = 20 μm; **j**, bar = 20 μm; **k**, bar = 1 mm.

Autopsy examination revealed no significant systemic pathology outside of the nervous system and the brain appeared macroscopically normal. Conventional histology revealed a marked loss of motor neurones (MN) from the anterior horns of the spinal cord. Residual MN contained occasional Bunina bodies (Figure 3**i,j**). Immunohistochemistry (IHC) for p62 revealed compact and skein-like neuronal cytoplasmic inclusions in MN throughout the spinal cord, in anterior horn and Clarke's column (Figure 3**a**,**b**) as well as in the hypoglossal nucleus of the medulla. IHC for p62 performed on the hippocampus, cerebellum as well as frontal and temporal neocortex did not show the ubiquitinated neuronal cytoplasmic inclusions that are characteristic of MND caused by hexanucleotide repeat expansions of *C9ORF72* (data not shown) 25–28. Compact neuronal cytoplasmic inclusions were seen in the motor cortex (Figure 3**d**). Glial cytoplasmic inclusions (GCI) were also evident (Figure 3**b**) in motor regions but not in the midbrain, neocortex or hippocampus. Neuronal intranuclear inclusions (NII) were not observed with p62 or conventional stains. IHC for TDP-43 revealed both normal nuclear labelling and neuronal and glial cytoplasmic inclusions of similar morphology and distribution to that seen with p62 IHC (Figure 3**e**–**g**). There was concomitant loss of nuclear TDP-43 in cells which contained cytoplasmic TDP-43 inclusions. IHC for CD68 in the spinal cord revealed a microglial reaction in the grey and white matter (Figure 3**k**). The dorsal columns were spared and the corticospinal pathways (crossed and uncrossed) were most severely affected. This tract degeneration was not apparent in conventional myelin stains. IHC for ANG revealed granular labelling of the neuronal cytoplasm as described elsewhere 23. The histological appearances suggest that this most probably corresponds to staining of lipofuscin (Figure 3**i**). This pattern of ANG staining was also seen in the spinal cord tissue from a neurologically healthy control, a case of *SOD1-*related ALS and a case of SALS (data not shown). Staining was also evident in blood leucocytes and ependymal cells, providing an internal positive control. IHC for α-smooth muscle actin labelled the smooth muscle layer of blood vessels strongly. There was no nuclear or cytoplasmic labelling of neurones or glia in the p.K54E *ANG* case. IHC for FUS showed normal, predominantly nuclear labelling without any neuronal cytoplasmic inclusions (Figure 3**h**), and βA4 amyloid was negative throughout motor system, midbrain and hippocampus. IHC for α-actinin-2 in skeletal muscle showed normal labelling of Z-discs (figure 3**l**).

## Discussion

Mutation screening of the *ANG* gene in a large cohort of ALS patients from Northern England identified a single mutation in a SALS case: c.232A>G that results in p.K54E amino acid change. This mutation was not detected in 278 controls from Northern England. A previous report identified this mutation in a German SALS patient, while it was absent from 616 controls 16. Taking into account all the previous reports, as summarized by van Es, the p.K54E mutation is absent from a total of 7946 controls 9,11,12,13,18. The ANG K54 residue is highly conserved in mammals and substitution of the lysine by glutamic acid is predicted to alter function and stability, by replacing the basic residue with an acidic one.

The clinical findings in our case were of a limb onset, rapidly progressive, predominantly lower motor neurone ALS phenotype in a 49-year-old male. In addition, the patient had reduced sural nerve sensory action potentials. This compares with the previously reported case with a p.K54E mutation which was also a male who had limb onset disease but at the much younger age of 28 years with a longer survival of 24 months and who also had frontal lobe dysfunction. There was no evidence of cognitive impairment in the case described in this report. Analysis of all *ANG*-related ALS cases where clinical details were available revealed that approximately 58% of cases are limb onset and 42% are bulbar (http://alsod.iop.kcl.ac.uk) 29.

Neuropathology findings have been reported in only one *ANG*-related ALS case previously, carrying a p.K17I substitution 23,30. A highlighted feature of this case was the presence of eosinophilic neuronal intranuclear inclusions in the pyramidal neurones of the hippocampus and frontal cortex that were immunoreactive for ubiquitin, p62 and α-smooth muscle actin. These were not detected in the p.K54E *ANG* case described in this report. The characteristic ALS neuropathology of TDP-43- and ubiquitin-positive neuronal and glial inclusions was present in both cases, while neither case showed any abnormalities of immunostaining for ANG. The p.K17I *ANG* case also showed marked loss of α-actinin-2 immunoreactivity in peroneal muscle which was not detected in our p.K54E *ANG* case. It also is interesting to note that in our current case p62/TDP-43-positive neuronal cytoplasmic inclusions were identified in Clarke's column. Neuronal cytoplasmic inclusions have been demonstrated with immunohistochemistry for p62 in SALS 31, phosphorylated neurofilament in *SOD1*-associated FALS 32 and FUS in *FUS*-associated FALS cases with basophilic inclusions 33.

While the neuropathological findings in our current case differ from the previous report, this is not surprising as findings from other ALS genes do not indicate that each gene has a unique pathological appearance. This is further supported by the finding of the p.K17I cases' characteristic neuronal nuclear alpha-actin inclusions neuropathology in cases which do not carry *ANG* mutations (Ansorge, pers. comm.). In addition, there is some debate as to whether p.K17I is pathogenic – this substitution is one of only two *ANG* nucleotide changes which have also been identified in controls from Europe 9,16,18. A recent study of 6471 ALS cases and 7668 controls concluded that these two variants, p.K17I and p.I46V, should be considered as polymorphisms, rather than pathogenic mutations 18.

The discovery of TDP-43 as the signature disease protein in pathological inclusions in SALS and in the majority of the related condition frontotemporal lobar degeneration with ubiquitinated inclusions (FTLD-U) has shed light on a possible common pathway leading to neurodegeneration in these conditions 34. However, an important question remains about how the other ALS-associated genes, with their varying neuropathological appearances, but clinically indistinguishable patterns of disease, fit into this pathway. It is known that the pathological inclusions in ALS cases associated with mutations in *SOD1* and *FUS* genes contain aggregated SOD1 and FUS proteins, respectively, rather than TDP-43 35,36. In the case of SOD1, this may be consistent with the toxic gain of function exhibited by the mutant protein, such that production of misfolded, mislocalized and inactive mutant protein exceeds the cells' protein degradation capacity resulting in formation of insoluble aggregates 37. Early cell models of ALS-related *FUS* mutants also suggest a possible toxic gain of function mechanism with increased cytoplasmic localization of the mutant protein 3. Similarly, evidence from mouse models overexpressing TDP-43 also suggests toxic cytoplasmic protein aggregation as a mechanism leading to neurodegeneration 38.

The mechanism of *ANG*-mediated neurodegeneration has not yet been elucidated. However, functional data from cell models suggest a role for haploinsufficiency. ALS-associated *ANG* mutants have markedly decreased ribonuclease activity and are unable to translocate to the nucleus, both of which are essential to normal ANG function 10,39. The mutant forms of ANG also have impaired neuroprotective ability 40, and are proposed to act through impaired inhibition of apoptosis via the phosphatidylinositol 3-kinase/protein kinase B (PI3K–AKT) signalling pathway 41, a mechanism also implicated in SOD1-related ALS 42. However, no functional link between ANG and TDP-43 has yet been demonstrated, although both are involved in distinct aspects of RNA processing 43. Further functional studies are required to elucidate the pathological mechanism of *ANG* mutations and their potential interaction with TDP-43.
